# *NAA10* p.(N101K) disrupts N-terminal acetyltransferase complex NatA and is associated with developmental delay and hemihypertrophy

**DOI:** 10.1038/s41431-020-00728-2

**Published:** 2020-09-24

**Authors:** Nina McTiernan, Harinder Gill, Carlos E. Prada, Harry Pachajoa, Juliana Lores, CAUSES study, Thomas Arnesen

**Affiliations:** 1 Department of Biomedicine, University of Bergen, N-5020 Bergen, Norway; 2 Department of Medical Genetics, Children’s and Women’s Health Centre of British Columbia, Vancouver, BC V6H 3N1 Canada; 3 Division of Human Genetics, Cincinnati Children’s Hospital Medical Center, 45229 Cincinnati, OH USA; 4 Department of Pediatrics, University of Cincinnati College of Medicine, 45229 Cincinnati, OH USA; 5 Centro de Medicina Genomica y Metabolismo, Fundacion Cardiovascular de Colombia, Floridablanca, Colombia; 6 Centro de Investigaciones en Anomalías Congénitas y Enfermedades Raras Universidad Icesi, Cali, Colombia; 7 Fundación Clínica Valle del Lili, Cali, Colombia; 8 Department of Medical Genetics, University of British Columbia, Vancouver, BC Canada; 9 Department of Biological Sciences, University of Bergen, N-5020 Bergen, Norway; 10 Department of Surgery, Haukeland University Hospital, N-5021 Bergen, Norway

**Keywords:** Mutation, Molecular biology, Disease genetics

## Abstract

Nearly half of all human proteins are acetylated at their N-termini by the NatA N-terminal acetyltransferase complex. NAA10 is evolutionarily conserved as the catalytic subunit of NatA in complex with NAA15, but may also have NatA-independent functions. Several *NAA10* variants are associated with genetic disorders. The phenotypic spectrum includes developmental delay, intellectual disability, and cardiac abnormalities. Here, we have identified the previously undescribed *NAA10* c.303C>A and c.303C>G p.(N101K) variants in two unrelated girls. These girls have developmental delay, but they both also display hemihypertrophy a feature normally not observed or registered among these cases. Functional studies revealed that *NAA10* p.(N101K) is completely impaired in its ability to bind NAA15 and to form an enzymatically active NatA complex. In contrast, the integrity of *NAA10* p.(N101K) as a monomeric acetyltransferase is intact. Thus, this *NAA10* variant may represent the best example of the impact of NatA mediated N-terminal acetylation, isolated from other potential NAA10-mediated cellular functions and may provide important insights into the phenotypes observed in individuals expressing pathogenic *NAA10* variants.

## Introduction

N-terminal (Nt) acetylation is a ubiquitous protein modification that pertains to ~80% of the human proteome [1]. Eight N-terminal acetyltransferases (NATs), named NatA to NatH, have been identified to date, whereof all except NatG are expressed in humans [2]. The cellular roles of Nt-acetylation are manifold and not fully understood, but some reported functions include regulation of protein complex formation, folding, degradation, subcellular localization, and membrane interactions [2–6]. NatA is the major NAT accounting for almost half of the Nt-acetylome due to its broad substrate specificity [1]. NatA is comprised of the catalytic subunit NAA10 and its binding partners NAA15, HYPK, and NAA50 (NatE) [7–11]. Binding of NAA10 to NAA15 ensures ribosomal anchoring and alters the substrate specificity of NAA10 to NatA specific substrates including small, polar amino acids [10, 12–14]. Moreover, NAA10 also exists as a monomer in the cell and is suggested to independently act as a lysine acetyltransferase (KAT) and noncatalytic regulator of diverse target proteins [2, 15–19]. *NAA10* is an essential gene and loss of function is lethal in model organisms such as *T. brucei*, *D. rerio*, *D. melanogaster*, and *C. elegans* [20–23]. In humans, NAA10 has been implicated in cancer signalling pathways both as a tumour suppressor and an oncoprotein, and is believed to have a regulatory role in cell proliferation and survival [24]. Furthermore, *NAA10* missense variants have in recent years emerged as causative of genetic disease, collectively known as *NAA10*-related syndrome [25]. This X-linked condition is associated with a broad spectrum of phenotypes including developmental delay (DD), intellectual disability (ID), and cardiac abnormalities [26]. This was first discovered in 2011, when a *NAA10* c.109T>C p.(S37P) variant was detected as the cause of Ogden syndrome (OMIM #300855) [27]. Affected boys had severe global developmental delay, craniofacial abnormalities, hypotonia and cardiac arrhythmia, and died within 16 months of age. Studies revealed that the *NAA10* c.109T>C p.(S37P) variant led to impaired NatA complex formation as well as decreased Nt-acetylation of NatA substrates in patient cells [27–29]. A *NAA10* splice-site variant c.471+2T>A was identified as one cause of of Lenz Microphthalmia Syndrome (OMIM #309800) in four males presented with anophthalmia, ID, developmental delay, and other malformations [30]. Popp et al. reported a boy and a girl carrying the *NAA10* variants c.319G>T p.(V107F), and c.346C>T p.(R116W) respectively, with severe ID, postnatal growth retardation, hypotonia, and behavioural anomalies [31]. Casey et al. described two brothers with ID, facial dysmorphism, scoliosis, and long QT who harboured a c.128A>C p.(Y43S) variant inherited from their mildly affected mother [32]. A recurrent missense variant, c.247C>T p.(R83C), has been identified in seven females with ID and developmental delay [26]. In all of these cases, this variant arose de novo, except for one case of maternal inheritance in which the female also had an affected brother who suffered a neonatal death. Furthermore, a missense variant affecting the same amino acid, c.248G>A p.(R83H), was recently detected in two unrelated boys with ID, developmental delay, and hypertrophic cardiomyopathy [33]. Missense variants affecting Arg83 are believed to impair Ac-CoA binding and cause reduced Nt-acetylation, resulting in the observed phenotypes [26, 33]. Three variants, c.384T>A p.(F128I), c.382T>A p.(F128L), and c.332T>G p.(V111G), have been reported in four females with ID and functional studies showed reduced Nt-acetylation caused by destabilisation of the NAA10 structure [26, 34]. Another three males displaying global DD, ID, and hypertrophic cardiomyopathy were found to harbour a c.215T>C p.(I72T) variant [35]. Finally, a recent international cohort presented 23 individuals with ten different *NAA10* variants [36]. Three of the variants have previously been described including the recurrent c.247C>T p.(R83C) variant, which in this study was found in 11 more individuals. Novel *NAA10* variants presented in the cohort include c.29A>G p.(D10G), c.32T>G p.(L11R), c.259G>T p.(A87S), c.311C>A p.(A104D), c.361C>G p.(L121V), c.440T>C p.(M147T), and a frameshift variant c.455_458del p.(Thr152fs) [36]. An overview of previously described *NAA10* variants is available in the Supplementary information (Supplementary Table S1). As the clinical spectrum associated with *NAA10* deficiency is expanding and new variants continue to emerge, there is currently limited overall understanding of the underlying disease mechanisms involved. Here we present two unrelated females harbouring two different genetic *NAA10* variants, c.303C>A and c.303C>G, which both encode the same *NAA10* p.(N101K) variant. The females display overlapping phenotypes including developmental delay, dysmorphic features, hemihypertrophy, and hearing loss. Functional studies suggest that this variant only impairs NatA activity and not monomeric NAA10 function.

## Materials and methods

### Construction of plasmid

A plasmid encoding the NM_003491.3 *NAA10* c.303C>A p.(N101K) missense variant was generated using the Q5 Site Directed Mutagenesis Kit (New England Biolabs, MA, USA) and pcDNA3.1/*NAA10*-V5-His vector as template. The forward and reverse primers used were 5′-TGATAGAGAAATTCAATGCCAAATATGTCTCCC-3′ and 5′-TGGCTCGAGAGGCCTGGT-3′ respectively, with an annealing temperature of 68 °C. The variant was confirmed by sequencing.

### Immunoprecipitation and Western blot analysis

Transfection of HeLa cells (ATTC, CCL-2) was performed using X-tremeGENE 9 DNA Transfection Reagent (Roche, Basel, Switzerland) as described in the instruction manual. The amount of DNA used for the different vectors were as follows: 10–12 µg of pcDNA3.1/*NAA10*-N101K-V5, 2.5–4 µg of pcDNA3.1/*NAA10*-WT-V5, 7.5–10 µg of pcDNA3.1/*NAA15*-*myc*-His or 10 µg of pcDNA3.1/*LacZ*-V5 (control-vector). 5–10 µg of empty pcDNA3.1/V5 plasmid was co-transfected with pcDNA3.1/*NAA10*-WT-V5 to attain equal amounts of DNA in the transfection mix. Transfected cells were harvested after two days of growth and lysed in 0.5–1 ml IPH lysis buffer (50 mM Tris-HCl pH 8.0, 150 mM NaCl, 5 mM EDTA, 0.5% NP-40, 1× complete EDTA-free protease inhibitor cocktail) for 15 min at 4 °C on a rotator. The cell lysates were centrifuged at 17,000 × *g* and 4 °C for 5 min and the supernatants collected. For immunoprecipitation (IP), the lysates were incubated with 1.5–4 µg of V5-tag antibody (Invitrogen #R960-25, CA, USA) or 3 µg NAA15 antibody [7] (BioGenes, Berlin, Germany) at 4 °C for 2 h on a rotator before adding 20–40 µl of washed Dynabeads Protein G (Invitrogen, CA, USA). After overnight incubation, the beads were washed three times in IPH lysis buffer. Then, the beads were either resuspended in 90 µl acetylation buffer (50 mM Tris-HCl pH 8.5, 1 mM EDTA, 10% Glycerol) to be used in a Nt-acetylation assay or in ×1 sample buffer to be analysed by Western blot. Samples were analysed using standard Western blot technique, and immunoblots were probed with V5-tag antibody (1:5000 dilution, Invitrogen #R960-25), NAA15 antibody (1:2000 dilution, BioGenes (named anti-NATH) [7]) and myc-tag antibody (1:2000 dilution, Invitrogen #R950-25). The ChemiDoc XRS+ system (Bio-Rad, CA, USA) coupled with Imagelab Software (Bio-Rad, CA, USA) was used to image and quantify protein bands.

### [^14^C]-Ac-CoA-based Nt-acetylation assay

To compare the intrinsic enzyme activity of V5-immunoprecipitated NAA10 WT-V5 and NAA10 N101K-V5, Nt-acetylation assays were performed as described previously [37]. In short, we used triplicate reactions containing 10 µl immunoprecipitated enzyme, 50 µM [14C]-Ac-CoA (PerkinElmer, MA, USA), 200 µM oligopeptide SESS_24_ (SESSSKSRWGRPVGRRRRPVRVYP) or EEEI_24_ (EEEIAALRWGRPVGRRRRPVRVYP) (BioGenes, Berlin, Germany), and acetylation buffer with a total volume of 25 µl. Oligopeptide was omitted in negative control reactions. Reactions were run for 30 min at 37 °C and 1400 rpm in a thermomixer. Afterwards, 23 µl of the supernatant was transferred onto P81 phosphocellulose filter discs (Millipore, MA, USA) which were subsequently washed three times in 10 mM HEPES buffer (pH 7.4) and air dried. Finally, the filter discs were submerged in 5 ml Ultima Gold F scintillation mixture (PerkinElmer, MA, USA) and the Nt-acetylated product was measured by a TriCarb 2900TR Liquid Scintillation Analyzer (PerkinElmer, MA, USA).

### Multiple sequence alignment and structural modelling

A multiple sequence alignment was generated using Clustal Omega [38] and illustrated by ESPript 3.0 [39]. The protein sequences are available in Supplementary Table S2. The structural analysis of human NatA (PDB ID: 6C9M) [40] was performed using PyMOL [41]. Acetyl-CoA was inserted in the hNatA structure through superimposition with the *S. pombe* NAA10 structure (PDB ID: 4KVX) [13] solved with acetyl-CoA bound.

### Exome sequencing

The family 1 proband participated in a translational research study (CAUSES Study) with institutional ethical approval from the University of British Columbia [H15-00092]. The family 2 proband participated in a rare diseases registry, with institutional ethical approval from Fundación Valle del Lili [01504]. Genomic DNA was isolated using standard techniques from the peripheral blood of family trios (proband, mother, and father). Each member of the trio had hybridization-based exome capture sequencing performed on an Illumina platform at Ambry Genetics (Aliso Viejo, CA, USA) for family 1 and at Agilent Technologies (Santa Clara, CA, USA) for family 2. A bioinformatics pipeline identified functional rare variants that were consistent with Mendelian patterns of inheritance as determined with the trio family structure. Sequencing reads were aligned to a reference human genome based on hg19 (ftp://hgdownload.cse.ucsc.edu/goldenPath/hg19/) using Bowtie2–2.2.6 [42]. Once mapped, Picard-tools-1.139 (http://broadinstitute.github.io/picard/) marked duplicate reads, and GATK-3.5-0 was applied for indel realignment [43]. Samtools-0.1.19 and bcftools-0.2 were used to call and filter single-nucleotide variants and indels [44]. SnpEff-4.1L [45] annotated Ensembl 75 transcripts [46] and identified functional variants, which were then annotated and filtered against in-house and public databases: dbSNP 144 [47], 1000G [48], ESP6500SI-V2 [49], and ExAC v0.3 [50]. A list of rare functional variants consistent with Mendelian inheritance patterns was annotated and prioritised using custom scripts and ANNOVAR 2015–06–17 [51] with diagnostically relevant information (e.g., OMIM [52], ClinVar [53], DECIPHER [54], LOVD 3.0 [55], NCBI gene summaries (ftp://ftp.ncbi.nlm.nih.gov/refseq/H_sapiens/RefSeqGene/), RVIS [56], and dbNSFP v3.0 [57]). Functional studies were undertaken after both clinical teams independently approached the functional analysis lab at the University of Bergen. The *NAA10* c.303C>G p.(N101K) and *NAA10* c.303C>A p.(N101K) variants were submitted to ClinVar (SCV001193440 and SCV001335306).

## Results

### Clinical report: individual 1

**Fig. 1:**
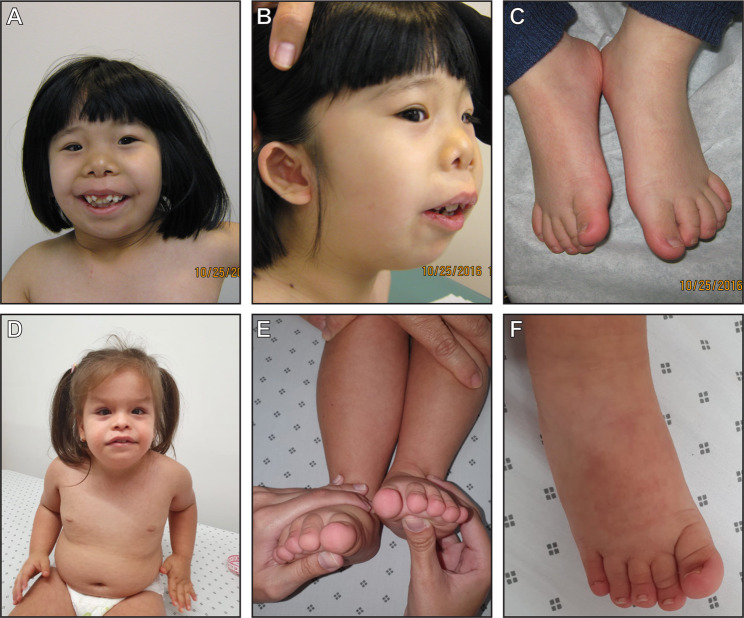
Patient photographs. Photographs of individual 1 showing dysmorphology (**a**), cup-shaped ears (**b**), hemihypertrophy (L>R) (**c**). Photographs of individual 2 showing dysmorphology (**d**), hemihypertrophy (R>L) (**e**) and broad hallux (**f**).

Individual 1 is a female with a de novo *NAA10* c.303C>A p.(N101K) (NG_031987.1 (NM_003491.3):c.303C>A, hg19:g.153197807G>T) variant who was referred to genetic evaluation at 5 years of age for global developmental delay, dysmorphic features, and left sided hemihypertrophy (Fig. 1a–c). She is the first child of her non-consanguineous parents and has a healthy younger sibling. The pregnancy was complicated by light per vaginam bleeding in the second trimester. She was born with a birth weight of 3.15 kg. Asymmetry of her body was noted in the neonatal period with the entire left side being larger than the right. She fed very slowly initially but did gain weight. Left sided hip subluxation was noted and surgically corrected with good result.

She failed her initial hearing test and on follow up was noted to have mild bilateral sensorineural hearing loss and wears hearing aids. Ophthalmic assessment revealed right posterior embryotoxon and anomalous optic nerves. An MRI of the brain was performed abroad and thought to be normal and we are awaiting repeat locally. Parents note delays in all areas of development. She has been diagnosed with moderate intellectual disability. Chromosome microarray, cardiac echo, and renal ultrasound were all normal. She did not meet the clinical criteria for Beckwith–Wiedemann syndrome (BWS) in that there was no evidence of neonatal hypoglycaemia, birthweight was on the 25th centile and she had no dysmorphic features of this condition. In addition, molecular testing for BWS returned negative. Renal ultrasound surveillance has remained normal. At the time of our first meeting she was noted to have significant micrognathia, short palpebral fissures and simple cup shaped ears (Fig. 1b). The hemihypertrophy is growing along with her, no sandal gap, thickening of the sole of the foot, vascular abnormalities, and lipomata. The left foot is 1.5 cm longer than the right foot (Fig. 1c). No other diagnosis for her asymmetry was diagnosed clinically. She has continued to grow along the 3rd centile for height and weight, and OFC has been −2SD below the mean. Exome sequencing identified a de novo variant of uncertain significance (VUS) in the *NAA10* gene: *NAA10* c.303C>A p.(N101K).

### Clinical report: individual 2

Individual 2 with a de novo *NAA10* c.303C>G p.(N101K) (NG_031987.1 (NM_003491.3):c.303C>G, hg19:g.153197807G>C) variant is a 4-year-old female referred to genetics evaluation for global developmental delay, dysmorphism, short stature, and right sided hemihypertrophy (Fig. 1d–f). Proband is the first child of non-consanguineous healthy parents of Colombian descent. She has one healthy sibling and no family history of congenital anomalies or intellectual disability. Mother was a healthy 29-year-old G2P1. Pregnancy was complicated by intrauterine growth restriction and prenatal imaging concerning for Dandy–Walker malformation. Individual was born at 36 weeks of gestational age via c-section. Birth weight was 1.53 kg (<3rd centile), length 40 cm (<3rd centile), and head circumference 29.5 cm (<3rd centile). The individual had poor respiratory effort requiring admission to the neonatal intensive care unit for 10 days. No additional hospitalizations or respiratory concerns. Hearing evaluation revealed severe bilateral hearing loss. Developmental history is remarkable for delayed walking (age 3) and speech (first words at age 4). BWS testing has not been performed as she does not meet the clinical criteria. Dysmorphology evaluation at age 4 years was remarkable for broad forehead, arched eyebrows, esotropia, broad columella, and full lips. She had joint hypermobility, short fingers with trident appearance, broad hallux, and the right foot was longer than the left foot (0.8 cm at last evaluation) (Fig. 1e, f). Growth parameters measured at last evaluation were weight was 17.15 kg (50th centile, 0.1 SD), height 90.8 cm (<3rd centile, −3.19 SD), and head circumference 49.5 cm (30th centile, 0.6 SD). Imaging evaluation included brain MRI that confirmed Dandy–Walker malformation and agenesis of the corpus callosum. Echocardiogram and renal ultrasound were normal. The girl has stereotypies and severe aggressive behaviour including biting and kicking caregivers. At last visit she was communicating using few words, her walk was more stable, and family reported that the she needed help with feeding, dressing, and was also not yet potty trained. Genetic workup included normal chromosomes (46, XX) and chromosomal microarray. Exome trio analysis was performed and this identified a de novo VUS in the *NAA10* gene: *NAA10* c.303C>G p.(N101K). This variant was not found in GnomAD exomes or genomes.

### Functional assessment of *NAA10* p.(N101K)

**Fig. 2:**
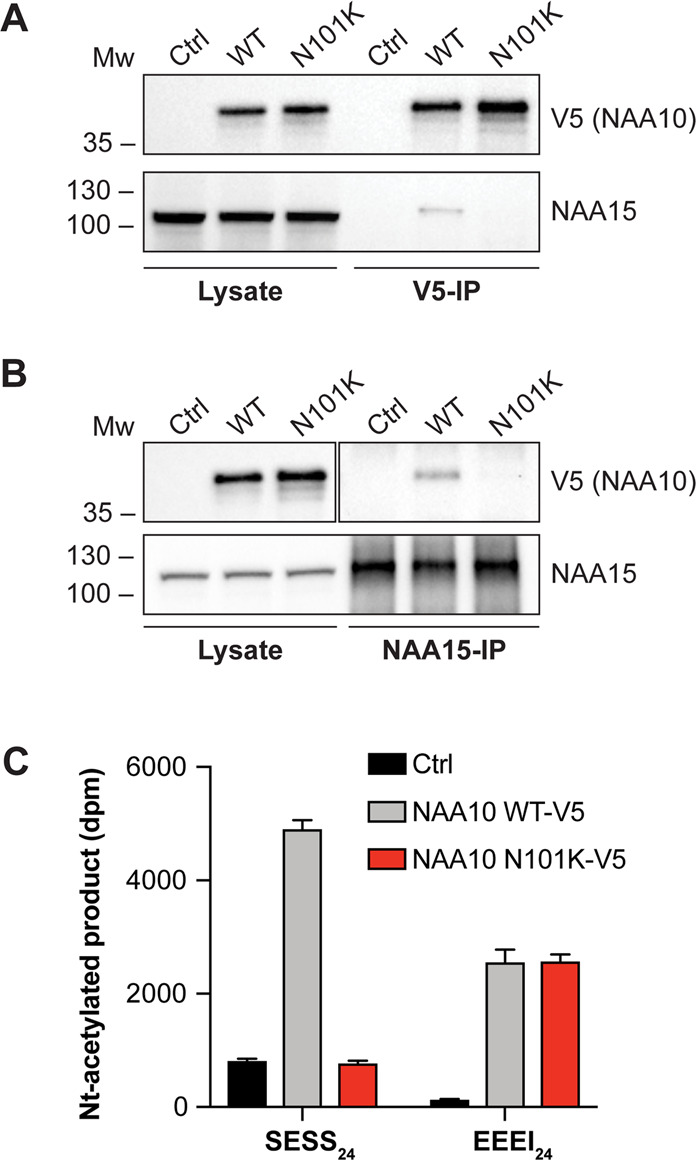
NatA complex formation and catalytic activity of immunoprecipitated NAA10 WT-V5 and NAA10 N101K-V5. NAA10 WT-V5 and NAA10 N101K-V5 were overexpressed in HeLa cells, immunoprecipitated by V5-tag antibody (**a**) or NAA15 antibody (**b**) and analysed by Western blotting. Densitometry analysis was performed to quantify NAA10-V5 and NAA15 bands. **c** Nt-acetylation assay displaying catalytic activity of immunoprecipitated NAA10 WT-V5 and NAA10 N101K-V5. The measured catalytic activity toward NatA substrate SESS_24_ and monomeric NAA10 substrate EEEI_24_ was normalised to the amount of immunoprecipitated NAA15 and NAA10-V5, respectively. Reaction mixtures either with immunoprecipitated β-gal-V5 or without peptide were used as negative controls to account for background signal. The IP and activity measurements were performed in three independent setups, each with three technical replicates per assay. One representative setup is shown.

In order to investigate the catalytic activity of NAA10 N101K in comparison to NAA10 WT, V5-tagged NAA10 was overexpressed in HeLa cells and immunoprecipitated using V5-tag antibody. The immunoprecipitates were used in Nt-acetylation assays and the amount of NAA10-V5 and co-immunoprecipitated NAA15 in the samples was determined by Western blot analysis (Fig. 2a). Interestingly, there was not detected any co-immunoprecipitation of NAA15 with the NAA10 N101K-V5 variant in contrast to NAA10 WT-V5, indicating that the missense variant hinders NatA complex formation. This observation was further supported by a reciprocal IP using NAA15 antibody, where only NAA10 WT-V5 co-immunoprecipitated with NAA15 (Fig. 2b). To exclude the possibility that NAA10 N101K-V5 does not bind NAA15 because it cannot compete with endogenous NAA10, we also simultaneously overexpressed both NAA15-myc and NAA10-V5 and performed V5-IP (Fig. S1). In agreement with the other IP experiments, NAA15-myc did not co-immunoprecipitate with NAA10 N101K-V5 while NAA15-myc readily formed a complex with NAA10 WT-V5.

The catalytic activity of NAA10 N101K-V5 and NAA10 WT-V5 was tested in an Nt-acetylation assay using the oligopeptides SESS_24_ and EEEI_24_, representing a NatA substrate and an in vitro monomeric NAA10 substrate, respectively (Fig. 2c, Supplementary Table S3). Since SESS_24_ is a NatA substrate, the catalytic activity toward this substrate was normalised to the amount of NAA15 in the immunoprecipitate, while the catalytic activity toward EEEI_24_ was normalised to the amount of NAA10-V5. As shown in Fig. 2c, NAA10 N101K-V5 has an abolished NatA activity toward SESS_24_ which is in accordance with the lack of co-immunoprecipitated NAA15 seen in the Western blot analysis (Fig. 2a). In contrast, the catalytic activity of NAA10 N101K-V5 toward EEEI_24_ was equal to that of NAA10 WT-V5, suggesting that the monomeric NAA10 catalytic function is not affected by the variant. Taken together, these results indicate that the *NAA10* c.303C>A and c.303C>G p.(N101K) variants are incapable of binding to NAA15, which results in abolished NatA catalytic activity, while monomeric *NAA10* c.303C>A and c.303C>G p.(N101K) catalytic activity remains intact.

### Multiple sequence alignment and structural analysis

**Fig. 3:**
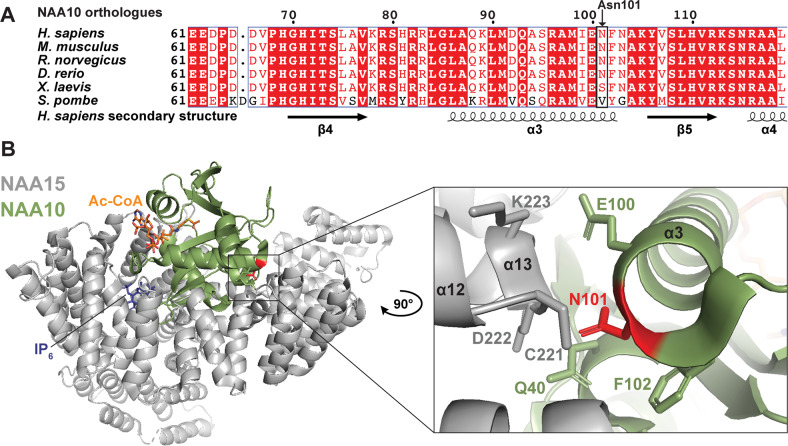
NAA10 multiple sequence alignment and NatA structural analysis. **a** Multiple sequence alignment of NAA10 orthologues from human, mouse, rat, zebrafish, frog, and yeast. Secondary structure was determined from hNatA structure (PDB ID: 6C9M) [40] and amino acid conservation is indicated by red colour. **b** Human NatA structure (PDB ID: 6C9M) [40] with the auxiliary subunit NAA15 (grey), the catalytic subunit NAA10 (green) and Ac-CoA and IP_6_ shown as orange and blue sticks, respectively. The structure was superimposed on Ac-CoA from the *S. pombe* NAA10 structure (PDB ID: 4KVX) [13]. The variant site Asn101 is coloured red. Close-up of Asn101 shows that it is located in NAA10 α3 helix with its side chain protruding toward NAA15.

NAA10 adapts the characteristic GCN5-related N-acetyltransfererase (GNAT) fold common for many acetyltransferases. The GNAT fold is a highly conserved structural domain comprising an Ac-CoA binding region, six or seven β-strands and four α-helices [58]. Asn101 is located within the conserved GNAT fold of NAA10, but a multiple sequence alignment of NAA10 orthologues revealed that the Asn101 residue itself is only semi-conserved across the species presented in Fig. 3a. Structural investigations of the hNatA structure (PDB ID: 6C9M) [40] showed that Asn101 is located in the α3 helix in close proximity to NAA15, with its polar side chain protruding toward the α12-loop-α13 segment of NAA15 (Fig. 3b). A structural analysis performed in PyMOL did not show any predicted interactions between the side chain of Asn101 and surrounding amino acids in neither NAA15 nor NAA10 (Fig. 3b).

## Discussion

In recent years, an increasing number of *NAA10* variants have been identified in both male and female individuals with varying degrees of phenotype severity [25]. In this study, we report two novel de novo genetic variants *NAA10* c.303C>A and c.303C>G p.(N101K) in two unrelated females with overlapping phenotypes including developmental delay, hemihypertrophy, hearing loss, and dysmorphic features. The significantly impaired function of *NAA10* p.(N101K) defined in our biochemical assays combined with the other features clearly classify these variants as pathogenic (class 5) according to ACMG guidelines [59]. X-inactivation patterns have previously been suggested to influence phenotype severity in female carriers of *NAA10* variants [26]. Due to the severe functional impairment of NatA activity of *NAA10* p.(N101K), and the necessity of NatA mediated Nt-acetylation for life in multicellular eukaryotes [20–23, 60], we speculate that the females harbouring the *NAA10* c.303C>A and c.303C>G p.(N101K) variants have skewed X-inactivation. However, this has not been experimentally tested.

Most of the previously characterised *NAA10* missense variants have been shown to reduce monomeric NAA10 NAT-activity in vitro after being ectopically expressed and purified [26, 27, 31–34]. Interestingly, a recent cohort study demonstrated that ectopically purified NAA10 variants displayed different effects on catalytic activity depending on whether it was present in the core NatA complex (NAA10-NAA15) or the trimeric NatA/HYPK complex (HYPK is a stable interactor of the NatA complex in vivo) [36]. For this reason, we believe that testing NAT-activity using immunoprecipitated NAA10 or NatA complexes from human cells, as performed herein, presents a more reliable method for predicting the catalytic consequences of *NAA10* variants in vivo. Due to the unresolved biochemical complexity of NAA10 and the plethora of downstream cellular phenotypes [2], a standardised assay to comparatively assess the full impact of a larger number of *NAA10* variants is not currently available.

Both IP of NAA10 N101K-V5 and reciprocal IP of NAA15 showed that NAA10 N101K-V5 does not bind NAA15 (Fig. 2a, b). Since this could be due to an inability of NAA10 N101K-V5 to compete with endogenous NAA10, a scenario avoided in patient cells expressing *NAA10* p.(N101K), NatA complex formation was also assessed in cells overexpressing both NAA10-V5 and NAA15-myc. Interestingly, even with an excess of NAA15-myc, NAA10 N101K-V5 was not able to bind NAA15 (Fig. S1). This strongly suggests that the missense variant completely eradicates NatA complex formation.

The in vitro Nt-acetylation activity assay displayed an abolished NatA catalytic activity of NAA10 N101K-V5, whereas the monomeric NAA10 catalytic activity appeared unaffected (Fig. 2c). In contrast to NAA10 N101K-V5, a portion of the NAA10 WT-V5 is complexed with NAA15 which exert little if any catalytic activity toward EEEI_24_ [14]. This could imply that the actual monomeric catalytic activity is slightly higher for NAA10 WT-V5 than the NAA10 N101K-V5 variant.

To understand why the *NAA10* c.303C>A and c.303C>G p.(N101K) variants hinder complex formation with NAA15, a sequence- and structural analysis was conducted. The multiple sequence alignment revealed that the Asn101 residue of NAA10 is not strictly conserved between orthologues (Fig. 3a). However, all the amino acids in position 101 are small and uncharged which suggest that lysine with its long, positively charged side chain is too dissimilar to be tolerated. Interestingly, Asn101 is located in the NAA10 α3 helix which is part of the contact surface with NAA15 and the side chain of Asn101 is protruding toward the NAA15 α12-α13 loop (Fig. 3b). A previous study that delineated the NAA10-NAA15 interactions of *S. pombe* NatA [13] reported that the NAA10 α1-loop-α2 region forms the most intimate interactions with NAA15, but the NAA10 α3 helix was also found to make intermolecular interactions that supplements the NAA10–NAA15 interface. It is plausible that the longer side chain and positive charge of lysine can cause steric hindrance/and or charge repulsion. Consequently, potentially important intermolecular interactions mediated by Asn101 and/or other residues in the NAA10 α3 helix could be disrupted and hinder optimal complex formation between NAA10 and NAA15.

Altogether, the data indicate that the *NAA10* c.303C>A and c.303C>G p.(N101K) variants abolish NatA complex formation and consequently also all NatA mediated N-terminal acetylation on the ribosome. Monomeric NAA10 has also been proposed to have NatA independent functions as a KAT catalysing lysine acetylation as well as a noncatalytic regulator of target substrates [2]. However, the NAA10 KAT activity toward some substrates has been disputed due to a lack of reproducibility [61]. The many cellular roles of NAA10 corroborates the complexity and challenge of defining the molecular mechanisms underlying clinical manifestations associated with *NAA10* deficiency. In the case of *NAA10* c.303C>A and c.303C>G p.(N101K), the variants do not seem to affect the monomeric functions of NAA10. Thus, the girls' phenotypes are most likely mediated via impaired NatA (NAA10-NAA15) Nt-acetylation activity, and not KAT, NAT, or noncatalytic roles of monomeric NAA10. Interestingly, the females harbouring the *NAA10* c.303C>A and c.303C>G p.(N101K) variants display hemihypertrophy, which has not previously been described for any individuals harbouring pathogenic *NAA10* variants. Thus this may be one such NatA-specific phenotype. In sum, *NAA10* p.(N101K) is the first variant reported to completely eradicate binding of NAA15 and it may uniquely reflect the functional impact of NatA.

## Supplementary information

Supplementary data

## References

[1] Arnesen T, Van Damme P, Polevoda B, Helsens K, Evjenth R, Colaert N (2009). Proteomics analyses reveal the evolutionary conservation and divergence of N-terminal acetyltransferases from yeast and humans. Proc Natl Acad Sci USA..

[2] Aksnes H, Ree R, Arnesen T (2019). Co-translational, post-translational, and non-catalytic roles of N-terminal acetyltransferases. Mol Cell..

[3] Shemorry A, Hwang CS, Varshavsky A (2013). Control of protein quality and stoichiometries by N-terminal acetylation and the N-end rule pathway. Mol Cell..

[4] Holmes WM, Mannakee BK, Gutenkunst RN, Serio TR (2014). Loss of amino-terminal acetylation suppresses a prion phenotype by modulating global protein folding. Nat Commun..

[5] Dikiy I, Eliezer D (2014). N-terminal acetylation stabilizes N-terminal helicity in lipid- and micelle-bound alpha-synuclein and increases its affinity for physiological membranes. J Biol Chem..

[6] Scott DC, Monda JK, Bennett EJ, Harper JW, Schulman BA (2011). N-terminal acetylation acts as an avidity enhancer within an interconnected multiprotein complex. Science..

[7] Arnesen T, Anderson D, Baldersheim C, Lanotte M, Varhaug JE, Lillehaug JR (2005). Identification and characterization of the human ARD1-NATH protein acetyltransferase complex. Biochem J..

[8] Arnesen T, Starheim KK, Van Damme P, Evjenth R, Dinh H, Betts MJ (2010). The chaperone-like protein HYPK acts together with NatA in cotranslational N-terminal acetylation and prevention of huntingtin aggregation. Mol Cell Biol..

[9] Arnesen T, Anderson D, Torsvik J, Halseth HB, Varhaug JE, Lillehaug JR (2006). Cloning and characterization of hNAT5/hSAN: an evolutionarily conserved component of the NatA protein N-α-acetyltransferase complex. Gene..

[10] Gautschi M, Just S, Mun A, Ross S, Rucknagel P, Dubaquie Y (2003). The yeast N(alpha)-acetyltransferase NatA is quantitatively anchored to the ribosome and interacts with nascent polypeptides. Mol Cell Biol..

[11] Mullen JR, Kayne PS, Moerschell RP, Tsunasawa S, Gribskov M, Colavito-Shepanski M (1989). Identification and characterization of genes and mutants for an N-terminal acetyltransferase from yeast. EMBO J..

[12] Magin RS, Deng S, Zhang H, Cooperman B, Marmorstein R (2017). Probing the interaction between NatA and the ribosome for co-translational protein acetylation. PLOS One..

[13] Liszczak G, Goldberg JM, Foyn H, Petersson EJ, Arnesen T, Marmorstein R (2013). Molecular basis for N-terminal acetylation by the heterodimeric NatA complex. Nat Struct Mol Biol..

[14] Van Damme P, Evjenth R, Foyn H, Demeyer K, De Bock P-J, Lillehaug JR (2011). Proteome-derived peptide libraries allow detailed analysis of the substrate specificities of N(alpha)-acetyltransferases and point to hNaa10p as the post-translational actin N(alpha)-acetyltransferase. Mol Cell Proteom..

[15] Lim J-H, Chun Y-S, Park J-W (2008). Hypoxia-inducible factor-1α obstructs a Wnt signaling pathway by inhibiting the hARD1-mediated activation of β-catenin. Cancer Res..

[16] Qian X, Li X, Cai Q, Zhang C, Yu Q, Jiang Y (2017). Phosphoglycerate kinase 1 phosphorylates Beclin1 to induce autophagy. Mol Cell..

[17] Ree R, Varland S, Arnesen T (2018). Spotlight on protein N-terminal acetylation. Exp Mol Med..

[18] Lee C-C, Peng S-H, Shen L, Lee C-F, Du T-H, Kang M-L (2017). The role of N-α-acetyltransferase 10 protein in DNA methylation and genomic imprinting. Mol Cell..

[19] Seo JH, Park J-H, Lee EJ, Vo TTL, Choi H, Kim JY (2016). ARD1-mediated Hsp70 acetylation balances stress-induced protein refolding and degradation. Nat Commun..

[20] Ingram AK, Cross GAM, Horn D (2000). Genetic manipulation indicates that ARD1 is an essential Nα-acetyltransferase in Trypanosoma brucei. Mol Biochem Parasit..

[21] Wang Y, Mijares M, Gall MD, Turan T, Javier A, Bornemann DJ (2010). Drosophila variable nurse cells encodes arrest defective 1 (ARD1), the catalytic subunit of the major N-terminal acetyltransferase complex. Dev Dyn..

[22] Sönnichsen B, Koski LB, Walsh A, Marschall P, Neumann B, Brehm M (2005). Full-genome RNAi profiling of early embryogenesis in Caenorhabditis elegans. Nature..

[23] Ree R, Myklebust LM, Thiel P, Foyn H, Fladmark KE, Arnesen T (2015). The N-terminal acetyltransferase Naa10 is essential for zebrafish development. Biosci Rep..

[24] Kalvik TV, Arnesen T (2013). Protein N-terminal acetyltransferases in cancer. Oncogene..

[25] Wu Y, Lyon GJ (2018). NAA10-related syndrome. Exp Mol Med..

[26] Saunier C, Støve SI, Popp B, Gérard B, Blenski M, Ahmew N (2016). Expanding the phenotype associated with NAA10-related N-terminal acetylation deficiency. Hum Mutat..

[27] Rope Alan F, Wang K, Evjenth R, Xing J, Johnston Jennifer J, Swensen, Jeffrey J (2011). Using VAAST to identify an X-linked disorder resulting in lethality in male infants due to N-terminal acetyltransferase deficiency. Am J Hum Genet.

[28] Myklebust LM, Van Damme P, Støve SI, Dörfel MJ, Abboud A, Kalvik TV (2015). Biochemical and cellular analysis of Ogden syndrome reveals downstream Nt-acetylation defects. Hum Mol Genet..

[29] Van Damme P, Støve SI, Glomnes N, Gevaert K, Arnesen T (2014). A Saccharomyces cerevisiae model reveals in vivo functional impairment of the ogden syndrome N-terminal acetyltransferase NAA10 Ser37Pro mutant. Mol Cell Proteom..

[30] Esmailpour T, Riazifar H, Liu L, Donkervoort S, Huang VH, Madaan S (2014). A splice donor mutation in results in the dysregulation of the retinoic acid signalling pathway and causes Lenz microphthalmia syndrome. J Med Genet..

[31] Popp B, Støve SI, Endele S, Myklebust LM, Hoyer J, Sticht H (2015). De novo missense mutations in the NAA10 gene cause severe non-syndromic developmental delay in males and females. Eur J Hum Genet..

[32] Casey JP, Støve SI, McGorrian C, Galvin J, Blenski M, Dunne A (2015). NAA10 mutation causing a novel intellectual disability syndrome with Long QT due to N-terminal acetyltransferase impairment. Sci Rep..

[33] Ree R, Geithus AS, Tørring PM, Sørensen KP, Damkjær M, Lynch SA (2019). A novel NAA10 p.(R83H) variant with impaired acetyltransferase activity identified in two boys with ID and microcephaly. BMC Med Genet..

[34] McTiernan N, Støve SI, Aukrust I, Mårli MT, Myklebust LM, Houge G (2018). NAA10 dysfunction with normal NatA-complex activity in a girl with non-syndromic ID and a de novo NAA10 p.(V111G) variant – a case report. BMC Med Genet..

[35] Støve SI, Blenski M, Stray-Pedersen A, Wierenga KJ, Jhangiani SN, Akdemir ZC (2018). A novel NAA10 variant with impaired acetyltransferase activity causes developmental delay, intellectual disability, and hypertrophic cardiomyopathy. Eur J Hum Genet..

[36] Cheng H, Gottlieb L, Marchi E, Kleyner R, Bhardwaj P, Rope AF (2019). Phenotypic and biochemical analysis of an international cohort of individuals with variants in NAA10 and NAA15. Hum Mol Genet..

[37] Drazic A, Arnesen T. [14C]-acetyl-coenzyme A-based in vitro N-terminal acetylation assay. In: Schilling O, editor. Protein terminal profiling: methods and protocols. New York, NY: Springer New York; 2017. p. 1–8. 10.1007/978-1-4939-6850-3_1 28315239

[38] Sievers F, Wilm A, Dineen D, Gibson TJ, Karplus K, Li W (2011). Fast, scalable generation of high-quality protein multiple sequence alignments using Clustal Omega. Mol Syst Biol..

[39] Robert X, Gouet P (2014). Deciphering key features in protein structures with the new ENDscript server. Nucleic Acids Res..

[40] Gottlieb L, Marmorstein R (2018). Structure of human NatA and its regulation by the huntingtin interacting protein HYPK. Structure..

[41] Schrödinger, LLC The PyMOL Molecular Graphics System, Version 2.3. 2019.

[42] Langmead B, Salzberg SL (2012). Fast gapped-read alignment with Bowtie 2. Nat Methods..

[43] McKenna A, Hanna M, Banks E, Sivachenko A, Cibulskis K, Kernytsky A (2010). The genome analysis toolkit: a MapReduce framework for analyzing next-generation DNA sequencing data. Genome Res..

[44] Li H, Handsaker B, Wysoker A, Fennell T, Ruan J, Homer N (2009). The sequence Alignment/Map format and SAMtools. Bioinformatics..

[45] Cingolani P, Platts A, Wang LL, Coon M, Nguyen T, Wang L (2012). A program for annotating and predicting the effects of single nucleotide polymorphisms, SnpEff: SNPs in the genome of Drosophila melanogaster strain w1118; iso-2; iso-3. Fly..

[46] Flicek P, Amode MR, Barrell D, Beal K, Billis K, Brent S (2014). Ensembl 2014. Nucleic Acids Res..

[47] Sherry ST, Ward MH, Kholodov M, Baker J, Phan L, Smigielski EM (2001). dbSNP: the NCBI database of genetic variation. Nucleic Acids Res..

[48] Auton A, Abecasis GR, Altshuler DM, Durbin RM, Abecasis GR, Bentley DR (2015). A global reference for human genetic variation. Nature..

[49] Exome Variant Server. NHLBI GO Exome Sequencing Project (ESP) (Seattle, WA) [http://evs.gs.washington.edu/EVS/].

[50] Lek M, Karczewski KJ, Minikel EV, Samocha KE, Banks E, Fennell T (2016). Analysis of protein-coding genetic variation in 60,706 humans. Nature..

[51] Wang K, Li M, Hakonarson H (2010). ANNOVAR: functional annotation of genetic variants from high-throughput sequencing data. Nucleic Acids Res..

[52] Online Mendelian Inheritance in Man, OMIM. McKusick–Nathans Institute of Genetic Medicine, Johns Hopkins University (Baltimore, MD) [https://omim.org/].

[53] Landrum MJ, Lee JM, Benson M, Brown GR, Chao C, Chitipiralla S (2018). ClinVar: improving access to variant interpretations and supporting evidence. Nucleic Acids Res..

[54] Firth HV, Richards SM, Bevan AP, Clayton S, Corpas M, Rajan D (2009). DECIPHER: database of chromosomal imbalance and phenotype in humans using Ensembl resources. Am J Hum Genet..

[55] Fokkema IF, Taschner PE, Schaafsma GC, Celli J, Laros JF, den Dunnen JT (2011). LOVD v.2.0: the next generation in gene variant databases. Hum Mutat..

[56] Petrovski S, Wang Q, Heinzen EL, Allen AS, Goldstein DB (2013). Genic intolerance to functional variation and the interpretation of personal genomes. PLoS Genet..

[57] Liu X, Wu C, Li C, Boerwinkle E (2016). dbNSFP v3.0: A one-stop database of functional predictions and annotations for human nonsynonymous and splice-site SNVs. Hum Mutat..

[58] Vetting MWS, de Carvalho LP, Yu M, Hegde SS, Magnet S, Roderick SL (2005). Structure and functions of the GNAT superfamily of acetyltransferases. Arch Biochem Biophys..

[59] Richards S, Aziz N, Bale S, Bick D, Das S, Gastier-Foster J (2015). Standards and guidelines for the interpretation of sequence variants: a joint consensus recommendation of the American College of Medical Genetics and Genomics and the Association for Molecular Pathology. Genet Med..

[60] Linster E, Stephan I, Bienvenut WV, Maple-Grødem J, Myklebust LM, Huber M (2015). Downregulation of N-terminal acetylation triggers ABA-mediated drought responses in Arabidopsis. Nat Commun..

[61] Magin RS, March ZM, Marmorstein R (2016). The N-terminal acetyltransferase Naa10/ARD1 does not acetylate lysine residues. J Biol Chem..

