# Supplementary material for: NAA10 p.(N101K) disrupts N-terminal acetyltransferase complex NatA and is associated with developmental delay and hemihypertrophy
Source: Eur J Hum Genet. 2020 Sep 24;29(2):280–8. doi: 10.1038/s41431-020-00728-2 (PMC7868364; doi:10.1038/s41431-020-00728-2)
Supplement: Supplementary file 1 — Supplementary data [file 41431_2020_728_MOESM1_ESM.docx]

**
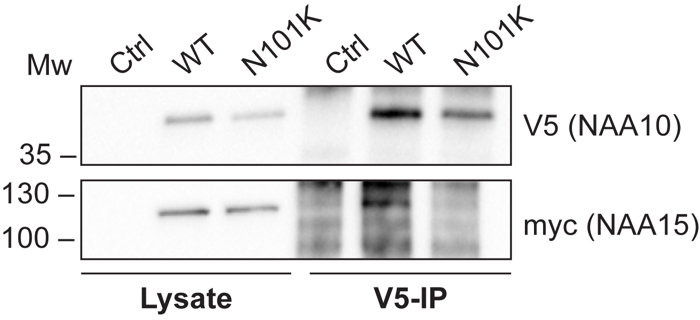
**

**Figure S1. Double transfection with NAA10-V5 and NAA15-myc followed by V5-immunoprecipitation.** HeLa cells were double transfected with NAA10 WT-V5 or NAA10 N101K-V5 and NAA15-myc before being subjected to anti-V5-immunoprecipitation, SDS-PAGE and Western blotting. The immunoprecipitation experiment was repeated three times and one representative experiment is shown.

**Table S1. Overview of *NAA10* variants and their effects**

| ***NAA10* variant** | **Individuals** | **Phenotype** | **Functional effect** | **References** |
| --- | --- | --- | --- | --- |
| p.(D10G)  c.29A>G | 1 male | Hypotonia, hearing loss, dysmorphic features, cardiac abnormalities, death in infancy | Reduced activity of NatA, reduced thermostability | Cheng et al., 2019 |
| p.(L11R)  c.32T>G | 1 female | Delayed motor  development, speech  delay, strabismus | Reduced activity of NatA, reduced thermostability | Cheng et al., 2019 |
| p.(S37P)  c.109T>C | 8 males | Global DD, craniofacial  abnormalities, aged  appearance, cardiac  arrhythmia, death in  infancy | Reduced activity of monomeric NAA10 and NatA, impaired  NAA15 and NAA50 interaction, reduced Nt-acetylation in patient fibroblasts, abnormal cell migration and proliferation | Myklebust et al., 2015;  Rope et al., 2011 |
| p.(Y43S)  c.128A>C | 2 males | ID, facial dysmorphism,  scoliosis, long QT | Reduced activity of monomeric NAA10 and NatA, reduced stability | Cheng et al., 2019;  Casey et al., 2015 |
| p.(I72T)  c.215T>C | 3 males | DD, ID, cardiac  abnormalities | Reduced activity of monomeric NAA10, reduced stability | Cheng et al., 2019;  Støve et al., 2018 |
| p.(R83C)  c.247C>T | 18 females  1 male | DD, ID, skeletal  abnormalities, cardiac  abnormalities | Reduced activity of monomeric NAA10 | Cheng et al., 2019;  Saunier et al., 2016 |
| p.(R83H) c.248G>A | 2 males | DD, ID, limited language development, hyperactivity,  and cardiac abnormalities | Reduced activity of monomeric NAA10 | Ree et al., 2019 |
| p.(A87S)  c.259G>T | 3 females | DD, ID, motor  development delay, non-verbal, hypotonia, hearing loss | Only clinically characterised | Cheng et al., 2019 |
| p.(A104D)  c.311C>A | 1 female | Global DD, ID, hypotonia, fine motor delay, short stature, hearing loss | Reduced activity of NatA, reduced thermostability | Cheng et al., 2019 |
| p.(V107F)  c.319G>T | 1 female | ID, abnormal muscle  tone, growth failure,  long QT, skeletal  abnormalities | Reduced activity of monomeric NAA10 | Popp et al., 2015 |
| p.(V111G)  c.332T>G | 1 female | ID, delayed motor and  language development | Reduced activity of monomeric NAA10, reduced stability | McTiernan et al., 2018 |
| p.(R116W)  c.346C>T | 1 female  1 male | ID, abnormal muscle  tone, growth failure,  skeletal abnormalities | Small reduction in activity of monomeric NAA10 | Popp et al., 2015 |
| p.(L121V)  c.361C>G | 2 females | DD, ID, motor  delay, non-verbal | Only clinically characterised | Cheng et al., 2019 |
| p.(F128L)  c.384T>A  c.384T>G | 3 females | ID, growth failure, delayed motor  development, abnormal  muscle tone | Reduced activity of monomeric NAA10, reduced stability | Cheng et al., 2019;  Saunier et al., 2016 |
| p.(F128I)  c.382T>A | 1 female | ID, growth failure,  abnormal muscle tone | Reduced stability, activity not assessed | Saunier et al., 2016 |
| p.(M147T)  c.440T>C | 1 female | DD, ID, limited speech, vision impairment | Reduced activity of NatA, reduced thermostability | Cheng et al., 2019 |
| p.(T152Rfs∗6)  c.455_458del | 1 male | Global DD, ID, hypotonia, growth delay, scoliosis, microphthalmia, syndactyly | Only clinically characterised | Cheng et al., 2019 |
| p.(E157fs45*)  c.471+2T>A | 8 males | ID (60% of cases),  anophthalmia or  microphthalmia, syndactyly, skeletal  and genitourinary  abnormalities | Truncated protein, activity not assessed, patient fibroblasts  have cell proliferation defects | Esmailpour et al., 2014 |

DD – developmental delay; ID – intellectual disability; QT – Q wave - T wave

**Table S2. NAA10 protein sequences used in multiple sequence alignment**

| Species | UniProt ID | Protein sequence |
| --- | --- | --- |
| *H. sapiens* | P41227 | MNIRNARPEDLMNMQHCNLLCLPENYQMKYYFYHGLSWPQLSYIAEDENGKIVGYVLAKMEEDPDDVPHGHITSLAVKRSHRRLGLAQKLMDQASRAMIENFNAKYVSLHVRKSNRAALHLYSNTLNFQISEVEPKYYADGEDAYAMKRDLTQMADELRRHLELKEKGRHVVLGAIENKVESKGNSPPSSGEACREEKGLAAEDSGGDSKDLSEVSETTESTDVKDSSEASDSAS |
| *M. musculus* | Q9QY36 | MNIRNARPEDLMNMQHCNLLCLPENYQMKYYFYHGLSWPQLSYIAEDENGKIVGYVLAKMEEDPDDVPHGHITSLAVKRSHRRLGLAQKLMDQASRAMIENFNAKYVSLHVRKSNRAALHLYSNTLNFQISEVEPKYYADGEDAYAMKRDLTQMADELRRHLELKEKGKHMVLAALENKAENKGNVLLSSGEACREEKGLAAEDSGGDSKDLSEVSETTESTDVKDSSEASDSAS |
| *R. norvegicus* | D3ZUQ2 | MNIRNARPEDLMNMQHCNLLCLPENYQMKYYFYHGLSWPQLSYIAEDENGKIVGYVLAKMEEDPDDVPHGHITSLAVKRSHRRLGLAQKLMDQASRAMIENFNAKYVSLHVRKSNRAALHLYSNTLNFQISEVEPKYYADGEDAYAMKRDLTQMADELRRHLELKEKGRHMVLSAMENKAENKGNVLLSSGEACREEKGLTAEDSGGDSKDLSEVSETTESTDVKDSSEASDSAS |
| *D. rerio* | Q7T3B8 | MNIRNARPEDLMNMQHCNLLCLPENYQMKYYFYHGLSWPQLSYIAEDENGKIVGYVLAKMEEDPDDVPHGHITSLAVKRSHRRLGLAQKLMDQASRAMIENFNAKYVSLHVRKSNRAALHLYSNTLKFQISEVEPKYYADGEDAYAMKRNLTQMADELQKPGVRLWGSEAPPSQDTSVTGLVEKLTVQDGEKEGDGDSGGESKEMSEVSEATESTDVKDSSSDS |
| *X. laevis* | Q7ZXW3 | MNIRNARPEDLMNMQHCNLLCLPENYQMKYYFYHGLSWPQLSYIAEDENGKIVGYVLAKMEEDPDDVPHGHITSLAVKRSHRRLGLAQKLMDQASRAMIESFNAKYVSLHVRKSNRAALHLYSNTLNFQISEVEPKYYADGEDAYAMKRDLTQMADEQLKKHLEIKEKSRPLSSIENKSDNRSRHVGDCCRDEKCMGNIGKQDLTEDSGDSKDVSEVSEATESTDVKDSSEASDSAS |
| *S. pombe* | Q9UTI3 | MDIRPARISDLTGMQNCNLHNLPENYQLKYYLYHAISWPMLSYVATDPKGRVVGYVLAKMEEEPKDGIPHGHITSVSVMRSYRHLGLAKRLMVQSQRAMVEVYGAKYMSLHVRKSNRAAIHLYRDTLQFDVQGIESKYYADGEDAYAMHKDFSTLKFDTPETNDELAKTVQSLALNN |

**Table S3. Catalytic activity of immunoprecipitated NAA10-WT-V5 and NAA10-N101K-V5.**

| Sample | DPM | | |
| --- | --- | --- | --- |
|  | Experiment 1 | Experiment 2 | Experiment 3 |
| WT + SESS | 5520 | 5032 | 6236 |
| WT + SESS | 4546 | 5090 | 6736 |
| WT + SESS | 4313 | 4782 | 6630 |
| WT + EEEI | 3203 | 2514 | 2188 |
| WT + EEEI | 2900 | 2455 | 2150 |
| WT + EEEI | 2621 | 2883 | 2522 |
| N101K + SESS | 883 | 787 | 915 |
| N101K + SESS | 898 | 817 | 933 |
| N101K + SESS | 795 | 888 | 871 |
| N101K + EEEI | 2504 | 2078 | 2573 |
| N101K + EEEI | 2626 | 2289 | 2535 |
| N101K + EEEI | 2323 | 2234 | 2252 |
| β-gal + SESS | 821 | 882 | 939 |
| β-gal + SESS | 817 | 913 | 923 |
| β-gal + SESS | 686 | 825 | 912 |
| β-gal + EEEI | 196 | 171 | 202 |
| β-gal + EEEI | 188 | 184 | 205 |
| β-gal + EEEI | 181 | 210 | 202 |
| WT + buffer | 40 | 87 | 44 |
| WT + buffer | 44 | 56 | 39 |
| N101K + buffer | 43 | 71 | 39 |
| N101K + buffer | 46 | 62 | 44 |

Nt-acetylation assay displaying catalytic activity of immunoprecipitated NAA10-V5 variants. Catalytic activity was measured towards NatA substrate SESS_24_ and monomeric NAA10 substrate EEEI_24_. Reaction mixtures either with immunoprecipitated β-gal-V5 or without peptide were used as negative controls to account for background signal. The immunoprecipitation and activity measurements were performed in three independent setups (Experiment 1-3), each with three technical replicates per assay. The three replicates of 10 µl immunoprecipitated enzyme, 200 µM synthetic oligopeptide SESS_24_ (SESSSKSRWGRPVGRRRRPVRVYP) or EEEI_24_ (EEEIAALRWGRPVGRRRRPVRVYP) (BioGenes), 50 µM [14C]-Ac-CoA (Perkin-Elmer) and acetylation buffer to a final volume of 25 µl were prepared. The samples were incubated at 37°C for 30 min at 1400 rpm shaking in a thermomixer. Thereafter, the beads were isolated and 23 µl of the supernatant transferred onto P81 phosphocellulose filter discs (Millipore). The filter discs were washed three times for 5 min in 10 mM HEPES buffer (pH 7.4) and air dried. Subsequently, the filter discs were added to 5 ml Ultima Gold F scintillation mixture (Perkin-Elmer) and the incorporated [14C]-Ac was measured by a Perkin-Elmer TriCarb 2900TR Liquid Scintillation Analyzer. DPM – disintegrations per minute.
